# Advancing Food Safety Surveillance: Rapid and Sensitive Biosensing Technologies for Foodborne Pathogenic Bacteria

**DOI:** 10.3390/foods14152654

**Published:** 2025-07-29

**Authors:** Yuerong Feng, Jiyong Shi, Jiaqian Liu, Zhecong Yuan, Shujie Gao

**Affiliations:** School of Food and Biological Engineering, Jiangsu University, Zhenjiang 212013, China; 18342628186@163.com (Y.F.); 2212418035@stmail.ujs.edu.cn (J.L.); 2212418018@stmail.ujs.edu.cn (Z.Y.); 2212418006@stmail.ujs.edu.cn (S.G.)

**Keywords:** biosensors, rapid detection, food safety monitoring, biorecognition elements, foodborne pathogenic bacteria

## Abstract

Foodborne pathogenic bacteria critically threaten public health and food industry sustainability, serving as a predominant trigger of food contamination incidents. To mitigate these risks, the development of rapid, sensitive, and highly specific detection technologies is essential for early warning and effective control of foodborne diseases. In recent years, biosensors have gained prominence as a cutting-edge tool for detecting foodborne pathogens, owing to their operational simplicity, rapid response, high sensitivity, and suitability for on-site applications. This review provides a comprehensive evaluation of critical biorecognition elements, such as antibodies, aptamers, nucleic acids, enzymes, cell receptors, molecularly imprinted polymers (MIPs), and bacteriophages. We highlight their design strategies, recent advancements, and pivotal contributions to improving detection specificity and sensitivity. Additionally, we systematically examine mainstream biosensor-based detection technologies, with a focus on three dominant types: electrochemical biosensors, optical biosensors, and piezoelectric biosensors. For each category, we analyze its fundamental principles, structural features, and practical applications in food safety monitoring. Finally, this review identifies future research priorities, including multiplex target detection, enhanced processing of complex samples, commercialization, and scalable deployment of biosensors. These advancements are expected to bridge the gap between laboratory research and real-world food safety surveillance, fostering more robust and practical solutions.

## 1. Introduction

Foodborne pathogens constitute critical risk determinants in food safety systems, with their contamination of food products precipitating significant public health crises and substantial economic losses [1,2]. In the United States alone, 31 major pathogenic agents are responsible for an estimated 9.4 million annual cases of foodborne illnesses, accounting for 55,961 hospitalizations and 1351 fatalities [3]. Among these, foodborne pathogenic bacteria rank as the second leading cause of foodborne illness, with notable culprits including *Salmonella*, *Escherichia coli* O157:H7(*E. coli* O157:H7), *Listeria monocytogenes* (*L. monocytogenes*), and *Staphylococcus aureus* (*S. aureus*). The clinical manifestations of these pathogens range from mild gastroenteritis to severe hemorrhagic colitis, septicemia, and even mortality, particularly endangering vulnerable populations such as infants, pregnant women, and the elderly [4,5,6,7,8,9]. In addition, foodborne diseases impose a considerable economic burden. In the United States alone, the estimated annual loss attributable to foodborne illnesses reaches approximately USD 17.6 billion [10]. Consequently, the development of rapid, sensitive, and highly specific detection technologies is of paramount importance for ensuring food safety [11].

Traditional methodologies for detecting foodborne pathogenic bacteria primarily encompass bacterial culture, biochemical identification, and molecular biology techniques, including polymerase chain reaction (PCR) and enzyme-linked immunosorbent assay (ELISA) [12]. While these techniques exhibit high diagnostic accuracy, they are often hindered by labor-intensive sample pretreatment protocols, prolonged detection times, and reliance on sophisticated laboratory equipment, rendering them inadequate for rapid on-site detection requirements [13]. In recent years, biosensor technology has emerged as a focal point of research in the field of foodborne pathogenic bacteria detection, owing to its remarkable advantages, including rapid response times, operational simplicity, high sensitivity, and suitability for field applications.

A biosensor is composed of three main components: a recognition element, a transducer, and a signal processing system. Among them, the recognition element determines the specificity of the sensor. Commonly used recognition elements include antibodies, aptamers, nucleic acids, enzymes, cell receptors, molecularly imprinted polymers (MIPs), and bacteriophages. The transducer is responsible for converting the biological signals generated by the interaction between the recognition element and the target pathogen into detectable physical or chemical signals—such as those based on electrochemical, optical, or mass-sensitive principles. The signal processing system amplifies, analyzes, and outputs these signals to enable qualitative or quantitative detection of pathogens [14,15,16].

**Figure 1:**
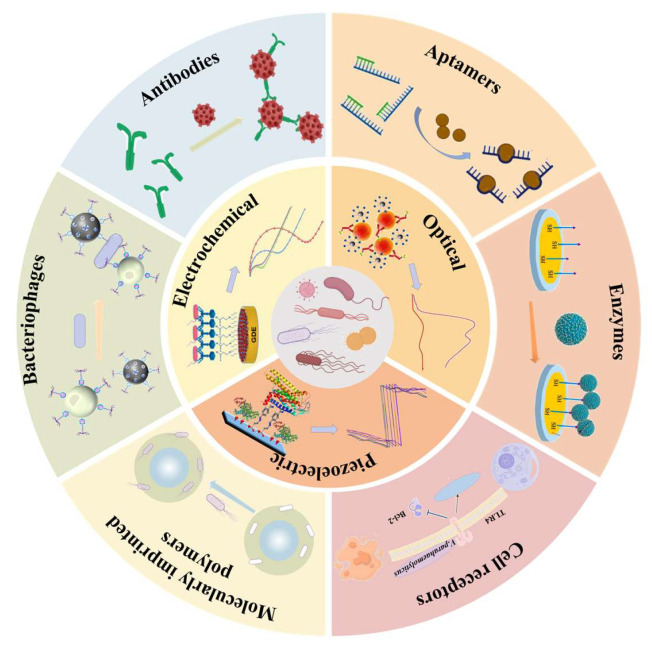
Schematic illustration of biosensor components for foodborne pathogen detection, including representative biorecognition elements and major transducer types.

This review systematically presents recent advances in biosensor-based detection of foodborne pathogenic bacteria, with a particular focus on the application and performance of diverse biorecognition elements and signal transduction mechanisms (Figure 1). Beyond summarizing design strategies, we emphasize the advantages and limitations of each technology, especially in complex food matrices. Compared with previous reviews that often focus on individual biosensor types or isolated components, this work provides a more integrated and application-oriented perspective. Special attention is given to practical indicators, such as detection limits, response times, anti-interference performance, and commercialization potential. By bridging fundamental biosensor design with real-world deployment challenges, this review aims to offer both theoretical insight and technical support for advancing next-generation food safety monitoring systems.

## 2. Biorecognition Elements for Monitoring Foodborne Pathogenic Bacteria

Biorecognition elements are the core components of biosensors, capable of specifically recognizing target analytes and triggering detectable signals. Their high selectivity significantly reduces the occurrence of false positives and false negatives during analysis, while their strong binding affinity to target molecules enables the capture of trace analytes, thereby greatly enhancing the sensitivity and accuracy of biosensors [17]. In addition, the stability of biorecognition elements ensures that biosensors maintain a high signal-to-noise ratio even in complex environments, making them widely applicable in fields such as food safety, environmental monitoring, and medical diagnostics [18,19]. Therefore, the selection of biorecognition elements and their interaction mechanisms with target analytes plays a decisive role in determining the overall performance of biosensors [20,21]. In this section, we provide a detailed discussion of biorecognition elements used for the detection of foodborne pathogenic bacteria, including antibodies, aptamers, nucleic acids, enzymes, cell receptors, MIPs, and bacteriophages (Table 1).

### 2.1. Antibodies

Antibodies, also known as immunoglobulins, are key molecules secreted by B lymphocytes in the immune system to combat foreign pathogens. Their fundamental structure consists of two identical heavy chains and two identical light chains, forming a characteristic Y-shaped configuration [22,23]. The variable region of the antibody, known as the Fab segment, contains the antigen-binding sites. These sites enable the antibody to specifically recognize and bind to target antigens, such as proteins, polysaccharides, or lipid molecules on the surface of pathogens, through a lock-and-key mechanism [24]. This highly specific binding capability establishes antibodies as one of the most critical recognition elements in biosensor applications.

**Figure 2:**
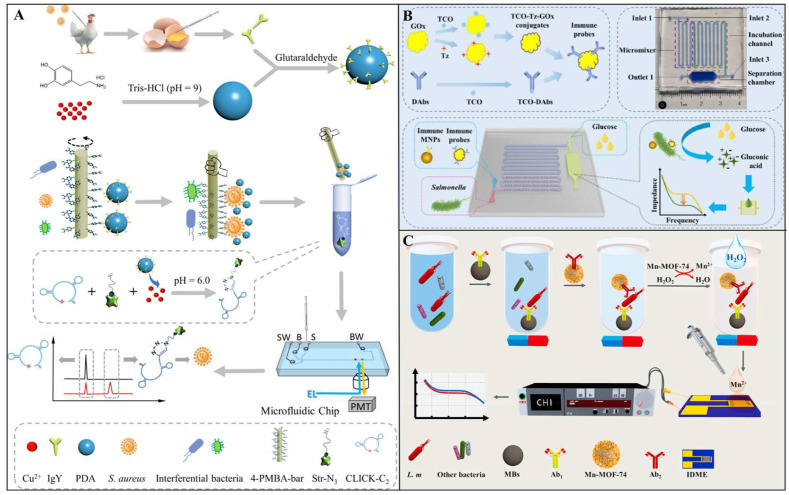
(**A**) Illustration of the proposed immunosensor for *S. aureus* detection [25]. Copyright 2022, Elsevier. (**B**) Schematic diagram of a microfluidic biosensor for rapid detection of *S. typhimurium* based on magnetic separation, enzymatic catalysis, and electrochemical impedance analysis [26]. Copyright 2022, Elsevier. (**C**) Schematic diagram of the sandwich model composed of Mn-MOF-74 impedance probe and immunomagnetic beads for the detection of *L. monocytogenes* [27]. Copyright 2021, Elsevier.

In biosensors, the core function of antibodies as recognition elements is to capture target pathogenic bacteria through their antigen-binding sites and subsequently trigger signal transduction processes. For instance, Wang et al. developed an ultrasensitive and simple microfluidic immunosensor based on stir bar enrichment and DNAzyme-assisted click reaction for the point-of-care detection of *S. aureus*. As shown in Figure 2A, *S. aureus* was first enriched using a 4-mercaptophenylboronic acid-functionalized stir bar, followed by the specific conjugation of yolk antibody (IgY) and copper-labeled polydopamine nanoparticles to the captured target. Under acidic conditions, the released Cu (II) effectively catalyzed the DNAzyme-assisted copper-catalyzed azide-alkyne cycloaddition (CuAAC) between alkyne group-labeled DNAzyme and streptavidin–biotin–azide, forming DNAzyme–streptavidin complexes. These complexes were then quantified via microfluidic chip analysis. Under optimized conditions, the immunosensor exhibited excellent detection performance for *S. aureus* within a range of 10 to 2.5 × 10^4^ CFU/mL, achieving a limit of detection (LOD) as low as 3 CFU/mL [25]. In another study, Liu et al. developed a microfluidic biosensor based on immunomagnetic separation, enzymatic catalysis, and electrochemical impedance analysis for rapid and sensitive detection of *Salmonella typhimurium* (*S. typhimurium*). As illustrated in Figure 2B, the biosensor captures antibody-modified magnetic nanoparticles (MNPs) and glucose oxidase (GOx)-conjugated detection antibody probes to form MNP–bacteria–probe sandwich complexes. During detection, glucose is enzymatically catalyzed by GOx into high-impedance hydrogen peroxide and low-impedance gluconic acid. The resulting impedance changes are measured using low-cost interdigitated microelectrodes and an electrochemical impedance analyzer to quantitatively determine the target bacteria. Under optimal conditions, the biosensor achieved a detection range of 1.6 × 10^2^ to 1.6 × 10^6^ CFU/mL for *S. typhimurium* within 1 h, with a low LOD of 73 CFU/mL. Its practical feasibility was further validated through spiked chicken meat samples [26]. The specificity of antibodies played a pivotal role in this system, ensuring high sensitivity and accuracy by enabling precise recognition and binding of the target bacteria.

The high specificity and affinity of antibodies enable them to effectively distinguish target bacteria from non-target bacteria in complex food matrices, significantly reducing the risks of false positives and false negatives. In a study, Wang et al. developed a novel impedance immunosensor based on a dual-antibody recognition strategy combined with the synergistic effect of a metal–organic framework (Mn-MOF-74) for the rapid and sensitive detection of *L. monocytogenes* in milk. As shown in Figure 2C, the dual-antibody recognition strategy achieves high specificity through a “capture antibody-target bacteria-detection antibody” sandwich structure: first, magnetic beads modified with capture antibody (MBs@Ab1) specifically isolate the target bacteria from the complex matrix; subsequently, Mn-MOF-74 modified with detection antibody (Mn-MOF-74@Ab2) binds to the target bacteria, forming a sandwich complex (MBs@Ab1-L. m-Mn-MOF-74@Ab2). Upon the addition of hydrogen peroxide (H_2_O_2_), Mn-MOF-74 releases Mn^2+^, significantly altering the impedance signal of a highly conductive gold interdigitated microelectrode, thereby enabling quantitative detection of the target bacteria. This sensor can complete the detection within 60 min, with LODs of 7.1 CFU/mL and 9.2 CFU/mL for *L. monocytogenes* in water and milk, respectively [27]. In complex food matrices, strategies like dual-antibody recognition and antifouling surface modifications have been employed to enhance selectivity and minimize background interference. However, the limitations of antibodies lie in their poor stability, as they may be inactivated by high temperatures, extreme pH, or proteases. To address this issue, researchers have developed single-chain variable fragments (scFv) or nanobodies, which have smaller molecular weights, simpler structures, and higher thermal stability, making them suitable for on-site detection [28,29].

### 2.2. Aptamers

**Figure 3:**
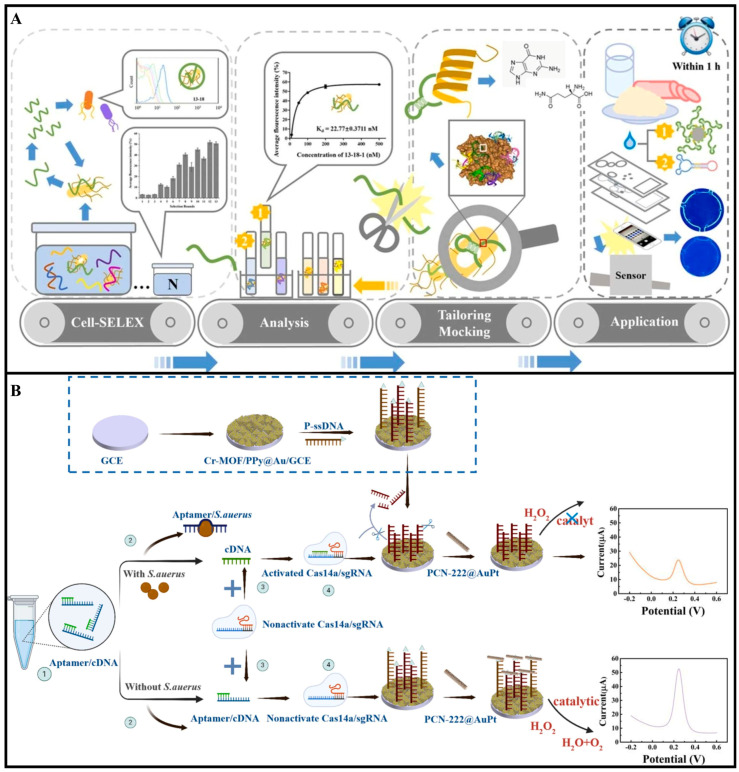
(**A**) Schematic diagram of a portable dual-aptamer microfluidic chip biosensor for *B. cereus* detection based on aptamer tailoring and dumbbell−shaped probes [33]. Copyright 2023, Elsevier. (**B**) Principle design diagram of CRISPR/Cas14a and PCN−222@AuPt nanozyme-based electrochemical biosensor for detection of *S. aureus* [40]. Copyright 2025, Elsevier.

Aptamers are single-stranded DNA or RNA molecules obtained through the Systematic Evolution of Ligands by Exponential Enrichment (SELEX) technique. They can fold into specific three-dimensional structures to bind surface proteins of pathogenic bacteria with high affinity and specificity. Their recognition mechanism relies on conformational matching and intermolecular interactions [30,31,32]. For instance, Zhou et al. developed a novel biosensor to address the challenges in detecting *Bacillus cereus* (*B. cereus*). As shown in Figure 3A, the researchers employed Cell-SELEX technology to screen phase-specific aptamers, optimized two high-performance aptamers, and revealed the interaction mechanism between repetitive guanine (G) bases in the aptamers and polar amino acids in the α-helix of surface proteins. Based on these findings, they designed dumbbell-shaped probes and a microfluidic chip-based biosensor, achieving ultrasensitive detection with an LOD of 9.27 CFU/mL within 1 h [33].

Aptamers exhibit robust thermal stability, acid–base resistance, and reusability, along with flexible chemical modification capabilities. Functional groups such as amino, thiol, or fluorescent moieties can be introduced at their termini to facilitate conjugation with nanomaterials for constructing signal amplification systems, which demonstrate strong anti-interference performance in complex samples. For instance, Yan et al. developed an ultrasensitive coreactant-free electrochemiluminescence (ECL) biosensor based on aptamer recognition for the detection of *S. aureus* in seafood. The study employed arginine/6-aza-2-thiothymine-modified gold nanoclusters (Arg/ATT-AuNCs) as luminophores, combined with an enzyme-mediated DNA walker and hybridization chain reaction (HCR) to achieve dual signal amplification. Upon specific recognition of the target bacteria by the aptamer, the walker strand was released, triggering the enzymatic cycling reaction, while the metal–organic framework (Zn/Co-MOF) served as the track for the DNA walker. In the HCR step, the enzymatically cleaved fragments induced a cascade reaction, significantly enhancing the conversion rate of H1. The biosensor demonstrated excellent linearity over the concentration range of 10^1^ to 10^9^ CFU/mL, with an LOD as low as 1.16 CFU/mL [34].

Beyond serving as standalone recognition elements, aptamers can be integrated with antibodies, enzymes, or CRISPR-Cas systems to construct multifunctional detection platforms, thereby further enhancing analytical performance and broadening the application scope. CRISPR-Cas systems, such as Cas12a, Cas13a, and Cas14a, have emerged as highly programmable nucleic acid-based recognition tools [35,36,37]. Guided by RNA sequences, these enzymes can specifically recognize target DNA or RNA and trigger collateral cleavage of nearby single-stranded nucleic acids, enabling sensitive and specific signal generation. Their excellent specificity, flexibility, and potential for attomolar-level sensitivity make them highly attractive for foodborne pathogen detection [38,39]. For example, Hui et al. developed an electrochemical aptasensor that combines the trans-cleavage activity of CRISPR/Cas14a with aptamer recognition, nanomaterial-modified electrodes, and nanozyme-catalyzed signal amplification for ultrasensitive detection of *S. aureus* (Figure 3B). The system employed Cr-MOF/PPy@Au composites to enhance conductivity and aptamer immobilization, while PCN-222@AuPt nanozymes provided peroxidase-like catalytic activity to amplify the electrochemical signal. The CRISPR-based cleavage mechanism ensured high specificity, achieving a detection limit as low as 10 CFU/mL over a wide dynamic range (5 × 10^1^ to 5 × 10^7^ CFU/mL) [40]. While challenges such as enzyme stability and cold-chain requirements remain, recent advances, including freeze-dried reagents and chip integration, are helping to move CRISPR biosensors toward practical, field-deployable food safety applications.

Despite their synthetic accessibility, high specificity, and ease of chemical modification, aptamers also face several critical limitations in practical biosensing applications. A major concern is their conformational instability in complex biological matrices or under extreme ionic conditions, which may impair binding efficiency. To improve structural robustness, researchers have developed chemically modified aptamers—such as those incorporating locked nucleic acids (LNAs) or phosphorothioate backbones—that exhibit enhanced nuclease resistance and stability [41]. Additionally, while aptamer selection via SELEX can be time-consuming and target-dependent, machine learning-assisted SELEX has recently emerged as an efficient approach to accelerate high-affinity aptamer identification. Another challenge is non-specific binding in food matrices, which can be mitigated through the use of structure-switching aptamer designs or ratiometric signal strategies that improve detection accuracy [42]. These advancements enhance the functional reliability of aptamer-based biosensors, supporting their broader application in foodborne pathogen detection.

### 2.3. Enzymes

Enzymes are biological macromolecules with highly efficient catalytic functions, predominantly proteins and, in rare cases, RNA. They accelerate specific biochemical reactions by lowering activation energy, and their activity relies on the high specificity of recognition and binding to substrates [43]. In pathogenic bacteria detection, enzymes serve as core recognition elements through their specific interactions with characteristic molecules on the surface of target bacteria or by indirectly capturing pathogens via enzyme-labeled antibodies/aptamers, thereby triggering signal amplification and transduction [44,45]. For example, Gu et al. developed a nanobody-horseradish peroxidase (Nb-HRP) sandwich ELISA for rapid, specific, and sensitive detection of *Salmonella Enteritidis* (*S. Enteritidis*) in milk samples [46].

While natural enzymes have demonstrated significant value in pathogenic bacteria detection due to their biological specificity, their application is limited by poor stability, low detection sensitivity, and complex labeling procedures. In recent years, nanozymes, as emerging artificial enzyme mimics, have successfully overcome these limitations through the precise design of inorganic nanostructures and enzyme-like active sites [47]. For instance, Li et al. developed an ultrasensitive electrochemical biosensor based on PtPd@PCN-224 nanozymes and the CRISPR/Cas14a system for detecting specific DNA sequences of *Burkholderia pseudomallei* (*B. pseudomallei*). The PtPd@PCN-224 nanozyme served as a signal amplification label, not only enhancing the electrochemical signal by catalyzing H_2_O_2_ reduction but also providing abundant active sites for nucleic acid probe assembly via Zr–O–P bonds. The CRISPR/Cas14a system further amplified the signal by recognizing target DNA sequences and triggering trans-cleavage of ssDNA. Leveraging the synergistic effects of nanozymes and the CRISPR system, the sensor achieved an ultra-low LOD of 12.8 aM, improving sensitivity by six orders of magnitude compared to traditional ELISA methods while accurately distinguishing non-target bacterial species [48].

However, significant challenges remain for enzyme-based recognition systems. Cross-reactivity between enzymes and non-target molecules may cause false positives, necessitating strategies like multi-enzyme cascades or composite probe designs to enhance specificity [49]. High production and purification costs, along with reliance on prokaryotic expression systems for certain enzymes, further limit their adoption in resource-limited settings. Although nanozyme immobilization techniques can partially improve stability, balancing enzyme activity preservation with detection throughput remains a critical technical challenge.

### 2.4. Cell Receptors

Cell receptors are functional proteins or glycoprotein complexes located on the cell membrane or within the cytoplasm. They specifically recognize and bind to particular ligands through spatial conformation, triggering downstream signaling cascades to mediate immune responses or metabolic regulation [50]. In pathogenic bacteria detection, the core mechanism of cellular receptors as recognition elements lies in mimicking natural host–pathogen interaction processes [51]. For example, the Toll-like receptor 4 (RpTLR4) in the Ruditapes philippinarum recognizes pathogen-associated molecular patterns (PAMPs) such as lipopolysaccharides or flagellin from *Vibrio parahaemolyticus* (*V. parahaemolyticus*), activating downstream signaling pathways and inducing hemocyte apoptosis. This receptor-mediated immune response not only elucidates the molecular mechanisms by which mollusks defend against Gram-negative bacteria but also provides biomimetic insights for developing cell receptor-based pathogen detection technologies. By simulating the specific binding between RpTLR4 and PAMPs, highly sensitive biosensors can be designed for the rapid detection of pathogenic bacteria in water or food [52]. Furthermore, Tomasek et al. revealed a novel immune evasion mechanism in which *E. coli* binds the host dendritic cell (DC) surface protein CD14 via the type 1 fimbrial tip protein FimH. The conserved domain of FimH interacts with the lipopolysaccharide-binding site of CD14, triggering dual immunosuppressive effects [53].

Despite their high specificity and ability to mimic natural host–pathogen interactions, cell receptor-based biosensors face several challenges that limit their broader adoption. One major limitation is the structural complexity and fragility of membrane-bound or recombinant receptors, which can result in reduced stability during sensor fabrication or storage. To address this, researchers have employed engineered receptor fragments or extracellular domains with simplified structures to retain binding activity while improving robustness. For example, Pérez et al. utilized a recombinantly expressed extracellular interleukin-5 (IL-5) receptor domain fused with Gly-His tags to enable stable immobilization on NiO nanoparticle-modified electrodes, demonstrating enhanced selectivity and electrochemical performance [54]. In addition, the relatively low abundance and high production cost of functional receptors remain significant bottlenecks. Solutions include the use of bacterial or yeast expression systems for scalable and cost-effective recombinant receptor production. Furthermore, the integration of receptor-mimetic materials, such as synthetic peptides or glycoprotein analogs, has been explored to replicate binding functions without relying on full-length native receptors. These advancements collectively contribute to improving the stability, affordability, and practical feasibility of receptor-based biosensors for foodborne pathogen detection.

### 2.5. Molecularly Imprinted Polymers

MIPs are synthetic materials designed based on biomimetic principles, with the core concept of creating “memory” cavities in polymer matrices that exhibit specific recognition capabilities [55,56,57]. In brief, during MIP synthesis, researchers select a surface protein or polysaccharide of pathogenic bacteria as the template. The template is complexed with functional monomers and immobilized via cross-linking polymerization, followed by template removal to leave cavities complementary to the template’s shape and chemical groups. These cavities enable high-selectivity rebinding of target molecules, akin to a “lock-and-key” mechanism, facilitating pathogen-specific recognition [58,59].

**Figure 4:**
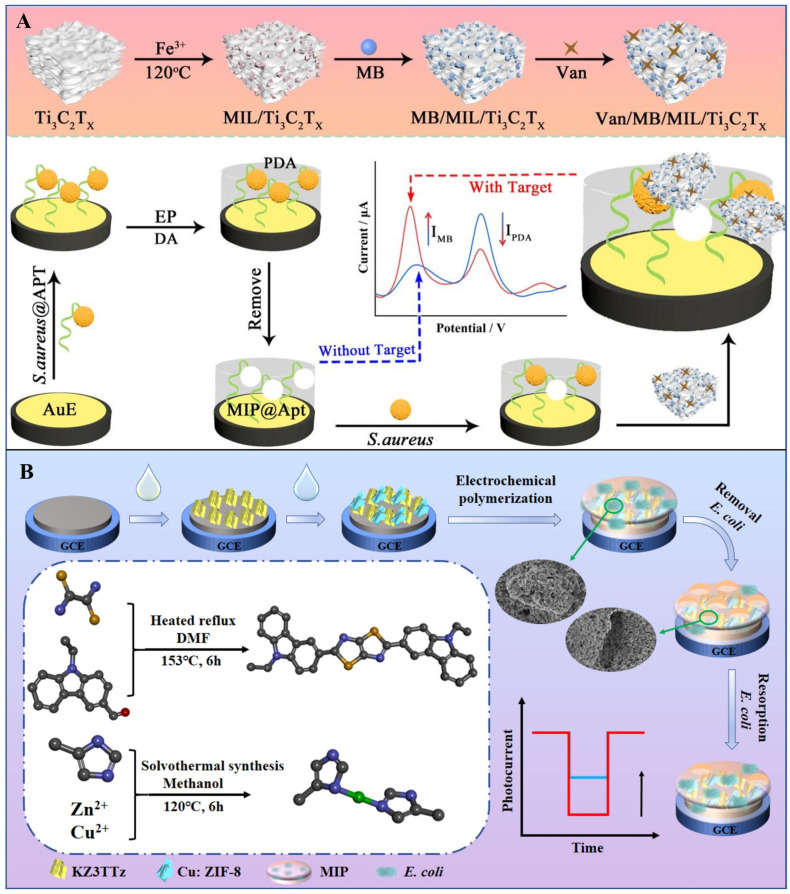
(**A**) Hybrid recognition-enabled ratiometric electrochemical sensing of *S. aureus* via in situ growth of MOF/Ti_3_C_2_Tx-MXene and a self-reporting bacterial imprinted polymer [61]. Copyright 2025, Elsevier. (**B**) Preparation process of MIP-PEC sensor and schematic illustration of its photoelectric detection mechanism for *E. coli* [62]. Copyright 2024, Elsevier.

The detection mechanism of MIPs relies on their ability to specifically bind surface biomarkers of pathogens. When pathogenic bacteria interact with an MIP-based sensor, surface molecules such as lipopolysaccharides or flagellin embed into the polymer cavities, forming stable complexes via hydrogen bonding, electrostatic interactions, or hydrophobic effects. This binding induces physical or chemical signal changes in the sensor, enabling quantitative or qualitative determination of pathogen presence and concentration [60]. For example, researchers developed a ratiometric electrochemical biosensor combining MIPs with aptamers (MIP@Apt complexes) for highly efficient detection of *S. aureus*. As shown in Figure 4A, the MIP@Apt complex serves as a dual-function recognition element: MIPs provide high-selectivity binding sites while acting as an internal reference (IR) probe for self-calibration. Additionally, a composite of metal–organic frameworks (MOFs) and transition metal carbonitrides (Ti_3_C_2_T_x_-MXene) is employed as a signal probe (SP). The sandwich structure formed between MIP@Apt and SP achieves ultrasensitive detection of *S. aureus* [61].

MIPs exhibit significant advantages over traditional antibody-based probes, including tolerance to high-temperature sterilization and extreme pH conditions, where antibodies often denature. Moreover, MIPs circumvent the need for biological culture systems, with synthesis typically completed in 3–4 days compared to the 4–6 weeks required for monoclonal antibody production. Their extended shelf life further enhances practicality for real-time pathogen monitoring. For instance, Wang et al. designed a stable molecularly imprinted photoelectrochemical (MIP-PEC) sensor by integrating polythiophene films with Cu:ZIF-8/KZ3TTz heterojunctions for *E. coli* detection (Figure 4B). The sensor demonstrated excellent linearity from 10^1^ to 10^8^ CFU/mL and an LOD of 4.09 CFU/mL [62].

However, the selective limitations and insufficient sensitivity of MIPs pose significant challenges. If the template molecules are not thoroughly eluted, residual template molecules may occupy the binding sites, leading to false positives. The sensitivity of MIPs is often inadequate, necessitating the integration of nanomaterials to amplify the signal. For instance, Narula et al. constructed an MIP layer on the surface of magnetic nanoparticles with a diameter of approximately 250 nm, using the specific marker protein A of *S. aureus* as the template molecule. In milk samples spiked with *S. aureus* (10^6^ CFU/mL), the MIPs demonstrated a cell recovery rate close to 100% [63].

### 2.6. Bacteriophages

Bacteriophages are a class of viruses that specifically infect bacteria. Their structure typically consists of genetic material encased within a protein capsid, with some phages possessing additional structures, such as tail fibers and sheaths [64]. Bacteriophages initiate infection by recognizing and binding to specific receptors on the bacterial surface, injecting their genetic material into the host, replicating within the cell, and ultimately lysing the host to release progeny phages. Owing to their high host specificity, bacteriophages are widely employed in bacterial detection, biotherapy, and ecological studies [65,66,67,68].

**Figure 5:**
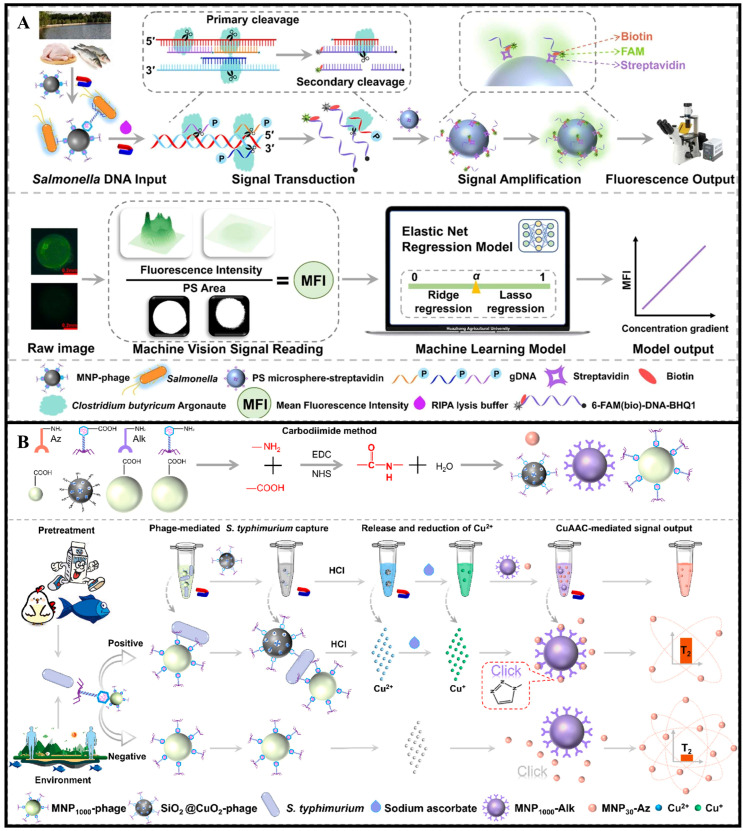
(**A**) Schematic diagram of machine vision-assisted Argonaute-mediated fluorescent biosensor for detection of live *Salmonella* in food [69]. Copyright 2024, Elsevier. (**B**) Schematic diagram of a CuO_2_@SiO_2_ nanoparticle-assisted click reaction-mediated magnetic relaxation biosensor for the rapid detection of *Salmonella* in food [70]. Copyright 2025, Elsevier.

The application of bacteriophages as recognition elements for pathogen detection primarily relies on their specific binding to target bacteria. Bacteriophages interact with bacterial surface receptors through tail proteins, exhibiting a “lock-and-key” matching mechanism that is highly specific to particular bacterial strains. In detection systems, bacteriophages can be integrated with various signal transduction methods to achieve bacterial identification. For instance, Zhao et al. developed a fluorescence biosensor for live *S. typhimurium* detection in food without requiring complex DNA extraction or amplification. As shown in Figure 5A, phage-mediated lysis of live bacteria releases DNA, which is specifically cleaved by Clostridium butyricum Argonaute (CbAgo) enzyme to generate guide sequences. These sequences trigger the targeted cleavage of fluorescent probes, releasing fluorescence signals amplified via streptavidin-coated microsphere enrichment and analyzed by machine vision algorithms. This system achieved an LOD of 40.5 CFU/mL, with a linear range of 50–10^7^ CFU/mL [69]. Another study by Zhao et al. established a phage-based magnetic relaxation switching (PCuMRS) sensor for rapid detection of live *S. typhimurium*. As shown in Figure 5B, phages conjugated to magnetic nanoparticles (MNPs) selectively captured target bacteria, while Cu^2+^-loaded CuO_2_@SiO_2_-phage nanoparticles released copper ions upon infection, triggering click chemistry-mediated aggregation of MNPs. This “phage recognition-chemical signal amplification-magnetic relaxation response” mechanism allowed the sensor to complete detection within 80 min, with a linear range of 10^2^–10^7^ CFU/mL and a quantification limit of 80 CFU/mL. Importantly, since phages only infect live bacteria, the sensor effectively avoided interference from dead bacterial residues, a common issue in traditional methods [70].

The advantages of bacteriophages as recognition elements include their high specificity, natural affinity, and stability. Phages exhibit strain-specific recognition, minimizing cross-reactivity. For example, Listeria phage A511 can distinguish *L. monocytogenes* from other *Listeria* species [71,72]. Their natural affinity eliminates the need for complex chemical modifications. For instance, phage T7 directly binds to the lipopolysaccharides of *E. coli* [73]. Additionally, bacteriophages are highly tolerant to extreme conditions such as high temperatures, pH variations, and organic solvents, making them suitable for complex sample analysis [74]. However, phage-based detection systems also have limitations. The lytic cycle of bacteriophages, which includes adsorption, replication, and lysis, can prolong detection time, hindering real-time analysis. For example, detection methods relying on lysis signals often require pre-culturing, making them slower than PCR-based techniques [75].

## 3. Transducers for Detecting Foodborne Pathogenic Bacteria

Transducers convert biorecognition events into measurable physical/chemical signals, dictating overall sensor sensitivity and practicability [76,77]. Recent advances in nanomaterial engineering, multimodal read-out, and AI-assisted analysis have further boosted their performance in complex food matrices [78,79]. In the following sections, we will discuss the principles, technological advancements, and application scenarios of mainstream transducers, with a focus on electrochemical, optical, and mass-sensitive transducers. We will analyze their design strategies, performance optimization pathways, and the practical challenges encountered in food sample analysis.

### 3.1. Electrochemical Transduction

Electrochemical transducers serve as core components of biosensors, functioning to transduce biorecognition events into quantifiable electrical signals (e.g., current, potential, or impedance), thereby enabling qualitative and quantitative detection of analytes [80,81,82,83]. In foodborne pathogen detection, these transducers leverage highly sensitive electrochemical response mechanisms to convert the specific binding between bacteria and biorecognition elements into measurable changes in electrical parameters, forming the technical foundation for rapid and precise assays [84]. Table 2 summarizes the key characteristics of electrochemical biosensors developed over the past five years for detecting foodborne pathogenic bacteria. As shown in Table 2, electrochemical biosensors have demonstrated remarkable analytical performance in detecting foodborne pathogenic bacteria. Many platforms achieve low detection limits that are comparable to or exceed the sensitivity of conventional PCR and ELISA methods. Moreover, the detection ranges reported span several orders of magnitude, indicating strong adaptability for both trace-level and high-load contamination scenarios.

#### 3.1.1. Voltammetric Transducer

**Figure 6:**
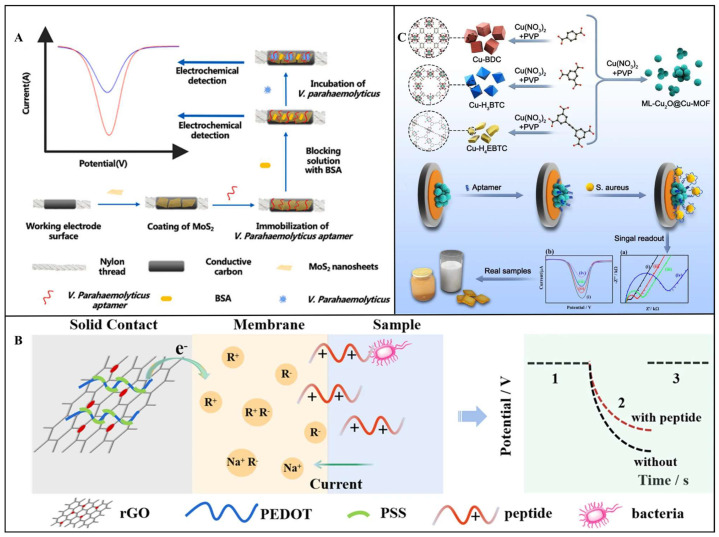
(**A**) Schematic representation of detection mechanism of thread−based microfluidic aptasensor for *V. parahaemolyticus* detection [91]. Copyright 2021, Elsevier. (**B**) Schematic diagram of an antimicrobial peptide−based potentiometric sensor for the detection of *S. aureus* [99]. Copyright 2021, Springer Nature. (**C**) Schematic diagram of the fabrication of *S. aureus* biosensor based on Cu−MOFs [104]. Copyright 2023, Elsevier.

Voltammetric sensors quantify targets by measuring current variations generated during electrochemical reactions, operating on electron transfer principles in redox processes. When target bacteria bind to biorecognition elements immobilized on electrode surfaces, they alter interfacial electron transfer efficiency, producing detectable current signals [85,86]. Common signal acquisition methods include CV, square wave voltammetry (SWV), and differential pulse voltammetry (DPV). Their core mechanisms involve two pathways: (1) Pathogen binding induces steric hindrance effects, impeding electron transfer of electroactive probes and causing current attenuation. (2) Enzyme labels or nanomaterials catalyze substrate reactions to generate electroactive species, significantly amplifying current responses [87,88,89]. For example, Li et al. developed an electrochemical biosensor based on the synergistic effect of CRISPR/Cas12a trans-cleavage activity and recombinase-aided amplification (RAA). In the presence of target *L. monocytogenes* DNA, RAA rapidly amplifies the target sequence, subsequently activating the trans-cleavage activity of Cas12a. This activation leads to the cleavage of surface-immobilized signal probes, resulting in a significant change in the electron transfer efficiency at the electrode interface. SWV is employed to accurately measure the redox peak current of electrochemical labels, enabling quantitative analysis of the target DNA. Under optimized conditions, this method achieves an LOD as low as 0.68 aM for genomic DNA and 26 CFU/mL for pure bacterial cultures [90]. In another study, Jiang et al. designed a microfluidic thread-based electrochemical aptamer sensor for rapid and highly sensitive detection of *V. parahaemolyticus* in seafood. As shown in Figure 6A, the sensor employs label-free aptamer immunodetection combined with the signal amplification mechanism of molybdenum disulfide (MoS_2_) nanosheets. MoS_2_ nanosheets, as high-surface-area conductive materials, significantly enhance the electrochemical signal response. When aptamers bind to *V. parahaemolyticus*, the bacteria impede electron transfer, reducing the characteristic peak current of MoS_2_ in DPV detection, with the reduction amplitude inversely correlated with the bacterial concentration. The method exhibits a linear range of 10–10^6^ CFU/mL, an LOD of 5.74 CFU/mL, and a detection time of 30 min [91].

Voltammetric sensors offer advantages such as high sensitivity, rapid response, and compatibility with portable devices, making them suitable for on-site screening. For instance, Yoon et al. developed a field detection system integrating a pretreatment device with a rapid electrochemical (REC) biosensor for *E. coli* gene detection in milk samples. The pretreatment device uses tetraethyl orthosilicate (TEOS)-coated microbeads combined with ultrasonic treatment to efficiently remove *E. coli* residues and extract DNA. By analyzing gene sequences of various foodborne pathogenic bacteria, a hypervariable region of *E. coli* DNA was selected as the biorecognition probe and immobilized on the electrode surface. To accelerate detection, alternating current electrothermal flow (ACEF) technology was introduced, reducing the binding time between the target and probe to 10 min. The REC biosensor achieved an LOD of 9.235 × 10^−5^ ng/μL for *E. coli* DNA fragments in milk samples, with an accuracy of 92.5%, a maximum error rate of 6.730%, and high selectivity in electrochemical performance evaluation [92]. However, voltammetric sensors also face notable limitations. Their susceptibility to interference from electroactive components in complex food matrices can lead to false-positive or false-negative signals, compromising detection reliability. Furthermore, the reliance on enzyme labels introduces operational limitations, as these biomolecules require stringent low-temperature storage to maintain activity stability, while the integration of signal amplification strategies adds layers of complexity to assay workflows. Compounding these issues, electrode surfaces are inherently vulnerable to nonspecific adsorption of biomolecules or contaminants, which progressively degrades sensor performance over repeated uses, thereby constraining their long-term practicality in resource-limited settings.

To overcome the above limitations and further push voltammetric sensors toward true point-of-care use, recent research has turned to paper-based electrochemical devices. By replacing conventional rigid electrodes with laser-patterned or screen-printed paper substrates, these platforms integrate hydrophilic sample conduits, on-paper filters, and low-cost carbon or metal inks into a single sheet that can be produced at minimal cost. The porous cellulose matrix naturally wicks complex food matrices, simultaneously performing debris removal and controlled reagent delivery, thereby mitigating matrix-induced interference and eliminating external pumps [93,94]. Moreover, wax-printed microzones confine the electrochemical cell to a few square millimetres, ensuring minimal reagent volumes and compatibility with handheld potentiostats or even smartphone audio-jack adapters. In a recent proof-of-concept, a paper-based analytical device (PAD) integrating screen-printed electrodes was used for the first time to quantify S. Typhimurium. Monoclonal antibodies against the pathogen were immobilized on the paper substrate to capture the bacteria, followed by the addition of a polyclonal antibody–colloidal gold conjugate (PA-AuNPs) to complete a sandwich immunocomplex. The formation of this complex produced a visible dark-red spot for rapid naked-eye screening, while the electrical conductivity measured between the electrodes provided accurate quantification. The sensor exhibited a logarithmic linear response from 10 to 10^8^ CFU mL^−1^ (R^2^ = 0.9882) with an LOD of 10 CFU mL^−1^, delivering results within 30 min. This simple, sensitive, and rapid PAD approach can be readily adapted to compact, portable readers for on-site food safety screening [95].

#### 3.1.2. Potentiometric Transducer

Potentiometric sensors operate based on steady-state changes in electrode surface potential governed by the Nernst equation, detecting voltage shifts caused by alterations in interfacial charge density or ion-selective membrane permeability upon target bacterial binding [96,97,98]. For example, Zhao et al. pioneered a novel label-free potentiometric detection strategy using charged antimicrobial peptides (AMPs) for *S. aureus* sensing. As shown in Figure 6B, a pulsed galvanostatic potentiometric sensor with exceptional stability was constructed using a reduced graphene oxide/poly(3,4-ethylenedioxythiophene): polystyrene sulfonate (rGO/PEDOT: PSS) solid-contact layer. A cationic AMP model was designed by introducing two arginine residues at the C-terminus, conferring high affinity and selectivity for *S. aureus*. Bacterial binding-induced surface charge density changes were directly quantified via chronopotentiometry without requiring labels or signal amplification. The sensor achieved a linear range of 10–1.0 × 10^5^ CFU/mL and an LOD of 10 CFU/mL under optimized conditions [99]. The advantages of potentiometric sensors lie in their simple device architecture, strong resistance to electrochemical interference, and suitability for long-term stable monitoring. However, they are significantly susceptible to environmental factors, requiring frequent recalibration to maintain accuracy.

#### 3.1.3. Impedimetric Transducer

Impedimetric sensors detect foodborne pathogenic bacteria by dynamically modulating electrochemical impedance at the electrode–solution interface during biorecognition events. This is achieved through a quantitative analysis of changes in charge transfer resistance (R_ct_) and double-layer capacitance (C_dl_) [100,101]. When pathogens bind to biorecognition elements immobilized on the electrode surface, their physical attachment obstructs electron transfer pathways or alters interfacial ion distribution, leading to significant impedance variations in high-frequency regions or low-frequency regions [102,103]. For example, Tian et al. engineered a multivalent copper-based metal–organic framework (ML-Cu_2_O@Cu-MOF) aptasensor for *S. aureus* detection. As shown in Figure 6C, ML-Cu_2_O@Cu-MOF nanospheres synthesized via mixed-ligand strategies featured defective crystalline structures integrating multiple copper valences (Cu^0^/Cu^+^/Cu^2+^) and Cu_2_O nanocrystals. These structural attributes enhanced aptamer anchoring efficiency and electrode conductivity. Real-time electrochemical impedance spectroscopy (EIS) monitoring of Rct changes caused by bacterial capture achieved a linear range of 10–1 × 10^8^ CFU/mL and an LOD of 2.0 CFU/mL [104].

While both voltammetric and impedimetric sensors involve electron transfer phenomena, they differ in their detection principles and signal interpretation. Voltammetric sensors directly measure current generated by redox reactions involving electroactive species, often requiring enzymatic or redox-active labels. In contrast, impedimetric techniques monitor changes in interfacial properties—such as charge transfer resistance and double-layer capacitance—without relying on direct redox reactions. This feature enables label-free detection and reduces susceptibility to electroactive contaminants commonly present in complex food matrices. Moreover, the ability to detect subtle physical and electrical changes at the electrode interface gives impedimetric sensors enhanced anti-interference performance and stability, making them particularly advantageous for foodborne pathogen detection. However, impedimetric analysis faces challenges in signal interpretation complexity. The requirement for equivalent circuit modeling to fit EIS data and extract characteristic parameters poses operational barriers for non-specialists. Additionally, high-frequency impedance measurements are vulnerable to interference from electrode surface roughness or nonspecific adsorption. To address the limitations of traditional impedimetric sensing, recent studies have explored the integration of CRISPR-Cas systems with impedimetric biosensing platforms. These hybrid systems leverage the highly specific nucleic acid recognition and trans-cleavage activity of Cas enzymes to induce measurable impedance changes [105,106].

**Table 2:** Summary of characteristics of electrochemical sensor methods used to detect foodborne pathogenic bacteria.

Electrochemical Methods	Targets	Range of Detection	LOD	Reference
DPV	*S. typhimurium*	10^1^ to 10^7^ CFU/mL	3 CFU/mL	[107]
CV	*S. typhimurium*	10^1^–10^5^ CFU/mL	10 CFU/mL	[108]
CV/DPV	*S. typhimurium*	1 to 1 × 10^5^ CFU/mL	23 CFU/mL	[109]
CV	*S. typhimurium*	6.7 × 10^1^ to 6.7 × 10^5^ CFU/mL	55 CFU/mL	[110]
EIS/CV	*S. aureus*	10^1^–10^5^ CFU/mL	0.28 CFU/mL	[111]
EIS/CV	*S. aureus*	0.01 fM–10 nM	10^−17^ M	[112]
EIS	*S. aureus*	10^2^ to 10^7^ CFU/mL	17 CFU/mL	[113]
DPV	*S. aureus*	5.0 × 10^0^–5.0 × 10^8^ CFU/mL	0.97 CFU/mL	[114]
EIS	*S. aureus*	10 to 10^7^ CFU/mL	7 CFU/mL	[115]
EIS/CV	*S. aureus*	12 to 6250 CFU/mL	3 CFU/mL	[116]
EIS/CV	*S. aureus*	10^2^ to 10^8^ CFU/mL	10 CFU/mL	[117]
CV	*S. aureus*	-	39 CFU	[118]
DPV/EIS	*L. monocytogenes*	1.9 × 10^1^ to 1.9 × 10^6^ CFU/mL	1.9 × 10^1^ CFU/mL	[119]
EIS/CV	*E. coli*	10^2^–10^9^ CFU/mL	10 CFU/mL	[120]
EIS	*E. coli* O157:H7	1.5 × 10^1^ to 1.5 × 10^5^ CFU/mL	4.0 CFU/mL	[121]
CV	*E. coli*	-	10^4^ CFU/mL	[122]
DPV/EIS	*S. aureus*	60 to 6 × 10^7^ CFU/mL	9 CFU/mL	[123]
EIS/CV	*V. parahaemolyticus*	10^1^ to 10^6^ CFU/mL	32 CFU/mL	[124]

### 3.2. Optical Transduction

Optical biosensors, a class of high-sensitivity detection technologies based on optical signal modulation, exhibit significant advantages in the rapid screening and precise analysis of foodborne pathogenic bacteria. Their core principle relies on interactions between light and biomolecules or target bacteria, enabling quantitative or qualitative analysis through the detection of optical signal changes. These sensors typically generate measurable optical signal variations upon specific binding between target bacteria and biorecognition elements, which are subsequently captured and analyzed by optical detection systems [125,126,127]. Based on the type of optical signals, they are primarily categorized into fluorescence sensors, colorimetric sensors, surface plasmon resonance (SPR) sensors, and surface-enhanced Raman scattering (SERS) sensors, each with distinct detection mechanisms and application scenarios [128,129]. The following sections systematically elaborate on the working principles, technical characteristics, and applications of these optical transducers in foodborne pathogen detection. Table 3 summarizes the key features of optical sensor-based methods for detecting foodborne pathogenic bacteria over the past five years. As summarized in Table 3, optical biosensors offer excellent analytical sensitivity and low detection thresholds, positioning them as competitive candidates alongside conventional methods like PCR. Dual-mode systems and ratiometric sensors further improve detection reliability by mitigating matrix interference and reducing false positives or negatives. While ELISA remains a common method for pathogen detection, it typically requires longer assay times and laboratory infrastructure. In contrast, the colorimetric and fluorescence biosensors summarized here offer rapid detection, visual output, and adaptability for field deployment, making them highly promising tools for real-world food safety monitoring.

#### 3.2.1. Fluorescent Transducer

Fluorescent sensors convert biorecognition events into measurable optical signals through fluorescent labels. This technology relies on characteristic emission light generated by fluorophores upon excitation, where variations in intensity, wavelength, or lifetime dynamically reflect the presence and concentration of target analytes [130,131,132,133]. In foodborne pathogen detection, fluorescent sensors typically employ antibodies, aptamers, or MIPs as recognition elements, coupled with labels such as quantum dots (QDs), organic dyes, or fluorescent proteins. Signal transduction is achieved via mechanisms including fluorescence resonance energy transfer (FRET), dynamic quenching effects (DQE), static quenching effects (SQE), and inner filter effects (IFE) [134,135,136,137]. Its notable advantages include high detection sensitivity, visualization capability, and dynamic monitoring potential. For instance, Ding et al. developed a magneto-fluorescent nanobiosensor by functionalizing receptor-binding protein 41 (RBP 41) from phage T102 onto magnetic beads (MBs) and quantum dot microspheres (QDMs). The sensor captures Salmonella via RBP 41-MBs and labels them with RBP 41-QDMs, forming MBs-RBP 41-bacteria-RBP 41-QDMs complexes. Fluorescence intensity quantification of these complexes enabled ultrasensitive detection of *Salmonella* with an LOD of 2 CFU/mL within approximately 1.5 h. The method demonstrated recovery rates of 87–119% in spiked food samples, confirming its practical applicability [138].

**Figure 7:**
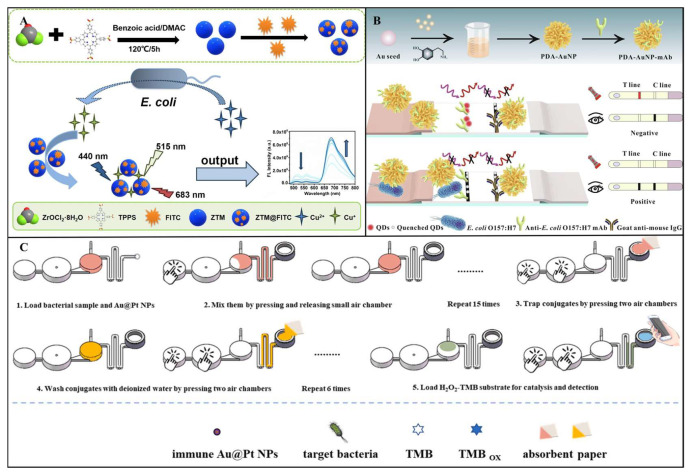
(**A**) Schematic diagram of the ratiometric fluorescence detection strategy for *E. coli* using ZTMs@FITC probes [139]. Copyright 2025, Springer Nature. (**B**) Schematic illustration of a novel dual-mode ICA biosensor based on PDA-AuNPs for the detection of *E. coli* O157:H7 [141]. Copyright 2023, Elsevier. (**C**) Schematic diagram of an integrated microfluidic chip-based colorimetric biosensor for the detection of *Salmonella* [153]. Copyright 2023, Elsevier.

Although single-mode fluorescence sensors exhibit remarkable performance, their detection stability is susceptible to pH fluctuations, temperature variations, and photobleaching effects. Additionally, the reliance on a single optical signal makes them prone to interference from instrumental parameter discrepancies. To address the limitations of single-mode fluorescence, the ratiometric fluorescence sensing mechanism has garnered significant attention. Ratiometric sensing typically relies on changes in dual-channel fluorescence signals, where the presence of target pathogens induces a quantifiable ratio change in the relative intensities of the two fluorescence signals, rather than merely an increase or decrease in a single channel’s fluorescence. For example, Zhang et al. developed a ratiometric fluorescent sensor based on fluorescein isothiocyanate (FITC)-labeled zirconium–tetraphenylporphyrin tetrasulfonic acid hydrate metal–organic frameworks (ZTMs@FITC) for highly sensitive detection of *E. coli*. As shown in Figure 7A, the sensor employs a dual-signal ratiometric detection system, utilizing the strong red fluorescence of ZTMs at 683 nm and the green fluorescence of FITC at 515 nm. The metabolic activity of *E. coli* reduces Cu^2+^ to Cu^+^, thereby restoring the fluorescence intensity of ZTMs, while the FITC fluorescence gradually decreases, resulting in a significant change in the F_683_/F_515_ ratio. The sensor enables rapid detection over a wide range of 1.0 × 10^1^ to 5.0 × 10^5^ CFU/mL, with an LOD as low as 6 CFU/mL, and shows a distinct fluorescence color change from yellow to red under 365 nm UV light [139].

Building on this, dual-mode fluorescent sensors have emerged, integrating fluorescence with electrochemical, colorimetric, or SERS signals to create cross-verification systems. For example, Gao et al. designed a ratiometric fluorescence-colorimetric dual-mode nanobiosensor using manganese dioxide nanosheets (MnO_2_ NSs) and boron-doped carbon dots (BCDs) for *S. aureus* detection. The sensor leverages MnO_2_ NSs-mediated fluorescence quenching and oxidation-driven signal amplification. The ratiometric fluorescence mode achieved an LOD of 9 CFU/mL, while the colorimetric mode reached 22 CFU/mL, with a linear range of 37–3.7×10^6^ CFU/mL. In real-world testing, recovery rates ranged from 90% to 102%, with relative standard deviations (RSDs) below 4.44% [140]. In another study, Shao et al. engineered a dual-mode immunochromatographic assay (ICA) using polydopamine (PDA)-functionalized gold nanoparticles (AuNPs) and ZnCdSe/ZnS quantum dots (QDs) for ultrasensitive *E. coli* O157:H7 detection. As shown in Figure 7B, the PDA-AuNPs exhibit broad-spectrum absorption for visible colorimetric signals, while their absorption spectrum overlaps with the excitation/emission spectra of QDs, enabling inner filter effect (IFE)-mediated fluorescence quenching. Target bacteria inhibit PDA-AuNPs/QDs interactions, restoring fluorescence signals. This dual-mode system achieved an LOD of 9.06 × 10^1^ CFU/mL in the fluorescence mode, representing a 46-fold sensitivity improvement over conventional AuNP-based ICA [141].

To address the practical challenge of mixed contamination by foodborne pathogenic bacteria, multi-array fluorescent sensors enable high-throughput parallel analysis through spatially resolved detection units. This technology employs fluorescent probes with distinct emission wavelengths to label specific recognition elements, achieving multi-channel synchronous detection via integration with microfluidic chips or fiber-optic arrays. For example, Liu et al. developed a multi-channel microfluidic chip (D-chip) based on a visual fluorescence distance readout mode for rapid and simultaneous detection of multiple bacteria. The chip achieves efficient separation and detection of mixed bacterial samples by pre-modifying specific channels with phages that capture different bacteria. During detection, target bacteria captured in their respective channels are labeled with aggregation-induced emission (AIE) fluorescent photosensitizer-conjugated antimicrobial peptides (AIE@AMPs), which enhance fluorescence signals upon binding. By measuring the distance of fluorescence emission channels, qualitative and quantitative analysis of bacteria is achieved. Under optimized conditions, the platform successfully detected *E. coli* O157:H7 (strain 44484) and two *S. typhimurium* strains (S. T 14028 and S. T 25928) within 30 min. The linear range for *E. coli* was 10^2^–10^6^ CFU/mL, with an LOD of 64 CFU/mL, while the linear range for both *S. Typhimurium* strains was 10^2^–10^6^ CFU/mL, with an LOD of 58 CFU/mL [142].

#### 3.2.2. Colorimetric Transducer

Colorimetric sensors are optical sensing technologies that detect targets through visible color changes. Their principle relies on interactions between targets and specific chemical or biological recognition elements, which trigger chromogenic reactions or alter the optical properties of nanomaterials, enabling qualitative or quantitative analysis via naked-eye observation or simple optical devices [143,144,145]. In foodborne pathogen detection, colorimetric sensors typically employ functionalized nanomaterials as signal carriers [146]. These materials exhibit pronounced localized surface plasmon resonance (LSPR) effects, where their absorption spectra undergo significant shifts due to aggregation states or surface chemical environment changes, manifesting as visible color variations in solutions or test strips [147,148]. For example, Song et al. developed a “capture-detection” tri-mode colorimetric biosensor based on a metal–organic framework (PCN-224)-anchored AuPt bimetallic nanozyme (AuPt/PCN-224, APP) and aptamer-functionalized magnetic microspheres (MBs) for ultrasensitive detection of *E. coli* O157:H7. The size-controlled APP demonstrated superior peroxidase-like activity, catalyzing the H_2_O_2_-TMB chromogenic reaction. Combined with the magnetic separation and enrichment capabilities of MBs, the sensor achieved a broad linear range of 10^1^–10^6^ CFU/mL and a low LOD of 10^1^ CFU/mL, exhibiting excellent performance in lake water, lettuce, and milk samples [149].

Colorimetric sensors are characterized by their simplicity, rapidity, and cost-effectiveness. Their ability to enable semi-quantitative detection through naked eye observation without complex instrumentation makes them particularly suitable for resource-limited field applications. Recent advancements in nanomaterial engineering, microfluidic integration, and intelligent data analysis platforms have further enhanced their performance [150,151,152]. For instance, Jin et al. developed a microfluidic-integrated colorimetric biosensor for rapid on-site *Salmonella* detection. As shown in Figure 7C, the sensor employs immunogold@platinum nanoparticles (Au@Pt NPs) to specifically label bacteria. A finger-actuated pneumatic mixer precisely controls reagent mixing, while nuclear track membranes with size-exclusion properties selectively retain bacteria–nanoparticle complexes. Upon *Salmonella* binding, the peroxidase-like activity of Pt catalyzes the H_2_O_2_-TMB chromogenic reaction. Color intensity analyzed via ImageJ software achieved an LOD of 168 CFU/mL within 25 min [153]. However, a critical challenge for colorimetric sensors lies in interference from complex food matrices that destabilize nanoparticles, potentially causing false signals. This necessitates the development of robust nanomaterials with enhanced anti-interference capabilities.

#### 3.2.3. SPR Transducer

SPR sensors are highly sensitive optical detection technologies based on the interaction between surface plasmon waves on metallic thin films and incident light. When light of a specific wavelength strikes the metal film at a critical angle, it excites SPR, causing a sharp decline in reflected light intensity. This critical angle is exquisitely sensitive to minute changes in the refractive index of the surrounding medium [154,155]. By immobilizing specific biorecognition elements on the sensor surface, the binding of foodborne pathogenic bacteria alters the surface mass and refractive index, thereby shifting the resonance angle. Real-time monitoring of this angular displacement enables qualitative and quantitative pathogen detection [156].

**Figure 8:**
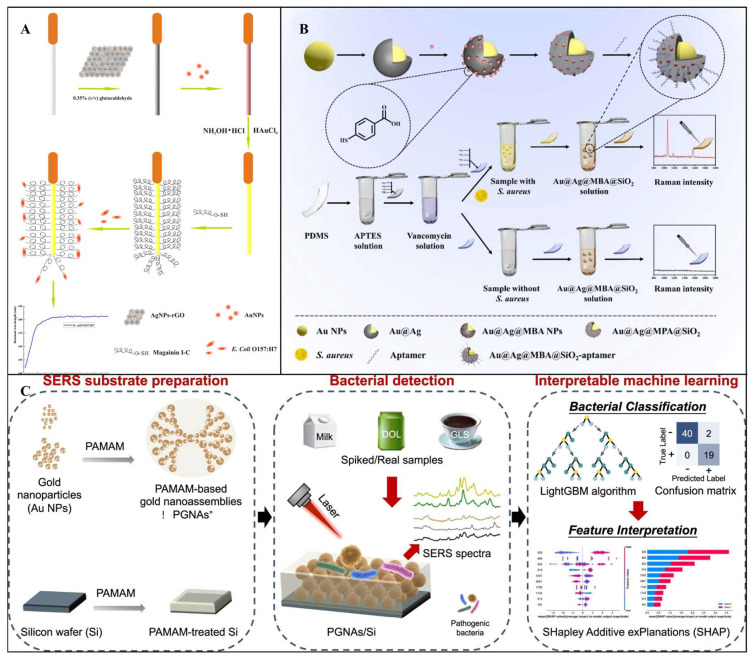
(**A**) Schematic diagram of a fiber optic SPR sensor based on antimicrobial peptides and AgNPs−rGO for the detection of *E. coli* O157:H7 [157]. Copyright 2018, Elsevier. (**B**) Schematic principle of the designed sandwich−type SERS biosensor for *S. aureus* detection [168]. Copyright 2022, Elsevier. (**C**) Schematic of a dendrimer−integrated SERS and incremental learning−inspired system for rapid detection of four pathogenic bacteria [170]. Copyright 2024, Elsevier.

SPR sensors are distinguished by their high sensitivity and real-time capabilities. For example, Zhou et al. developed a fiber-optic surface plasmon resonance (FOSPR) biosensor for detecting pathogenic *E. coli* O157:H7 in water and juice. As shown in Figure 8A, the sensor employs the antimicrobial peptide Magainin I as a specific recognition element, combined with signal amplification from silver nanoparticle-reduced graphene oxide (AgNPs-rGO) nanocomposites, significantly enhancing detection performance. The core principle relies on SPR sensitivity to interfacial refractive index changes: after immobilizing AgNPs-rGO nanocomposites and coating a gold film on the fiber-optic surface, incident light excites surface plasmon waves, generating an SPR absorption peak. When Magainin I captures target bacteria, interfacial mass changes cause a redshift in the SPR absorption peak wavelength, enabling quantification through monitoring the shift magnitude. The sensor exhibited a linear range of 1.0 × 10^3^–5.0 × 10^7^ CFU/mL, with an LOD as low as 500 CFU/mL, and demonstrated high specificity and stability in juice samples [157].

However, several challenges continue to hinder the widespread application of SPR sensors. The high cost of instrumentation and the complexity of surface functionalization procedures limit their accessibility, particularly in resource-limited settings. Furthermore, nonspecific adsorption of proteins, lipids, or other matrix components in complex food samples can result in signal interference and false-positive results, thereby reducing detection accuracy.

#### 3.2.4. SERS Transducer

SERS sensors are ultra-sensitive analytical technologies based on the amplification of Raman signals through specific nanostructures or material surfaces, enabling trace-level molecular detection [158,159,160]. Raman scattering arises from inelastic light–molecule vibrational interactions, where frequency shifts in scattered light reflect molecular vibrational energy levels, serving as unique “fingerprint spectra” [161,162]. However, conventional Raman scattering suffers from inherently weak signals, limiting practical applications. SERS overcomes this limitation by leveraging localized surface plasmon resonance (LSPR) effects in metallic nanostructures, which amplify electromagnetic fields near the surface, enhancing Raman signals by factors of 10^6^ to 10^12^ [163]. This is achieved by adsorbing target molecules onto nanostructured substrates; incident laser excitation induces localized electromagnetic field enhancement, coupling with molecular vibrational modes to generate intensified Raman signals. These signals enable specific identification of molecular species, concentrations, and even structures, facilitating highly sensitive detection of trace analytes [164,165,166]. In foodborne pathogen detection, SERS sensors identify bacteria-specific biomolecules or their labeled Raman signatures combined with targeted probes for qualitative and quantitative analysis [167].

SERS-based detection of foodborne pathogenic bacteria mainly relies on two strategies: one is the direct detection of the bacteria’s own molecular “fingerprint spectrum”; the other is an indirect detection mode, in which functionalized SERS probes bind to the target bacteria, resulting in detectable signal changes. For example, Wei et al. proposed a SERS biosensor based on a signal-amplifying sandwich system for the detection of *S. aureus*. As shown in Figure 8B, the study employed SiO_2_-coated Au@Ag core–shell nanoparticles as the SERS substrate and utilized vancomycin (Van) and polydimethylsiloxane (PDMS) modification for efficient target bacteria capture. The results demonstrated that the SiO_2_-coated Au@Ag nanoparticles exhibited strong and stable SERS responses, while the Van–PDMS combination significantly improved bacterial capture efficiency and simplified the separation process. In the presence of the target bacteria, a sandwich-like composite structure was formed, and the Raman signal was enhanced through a synergistic amplification effect. Under optimized conditions, the sensor exhibited good linearity over a wide dynamic range from 38 to 3.8 × 10^7^ CFU/mL, with an LOD as low as 2 CFU/mL [168].

SERS sensors excel in their “fingerprint” specificity, enabling the differentiation of pathogen serotypes [169]. For example, Qiu et al. developed a novel SERS platform integrating polyamidoamine (PAMAM)-functionalized gold nanoassemblies (PGNAs/Si) with class-incremental learning (CIL). As shown in Figure 8C, the dendritic nanostructure of PGNAs/Si enhanced sensitivity and reproducibility, achieving an LOD of 10 CFU/mL across diverse matrices. A LightGBM-based CIL model, combined with SHapley Additive exPlanations (SHAP) for feature selection, classified four pathogens with over 93.44% accuracy [170].

Despite their excellent sensitivity, SERS biosensors face several challenges, including poor reproducibility in nanostructured substrate fabrication, signal variability, and interference from food matrix components such as proteins and lipids. Spectral overlap further complicates multiplex detection. To overcome these issues, standardized substrate fabrication methods, antifouling surface modifications, and advanced data analysis techniques—such as machine learning and spectral deconvolution—have been applied [171,172,173]. These strategies significantly improve the stability, selectivity, and applicability of SERS platforms in complex food environments.

**Table 3:** Summary of characteristics of optical sensor methods used to detect foodborne pathogenic bacteria.

Optical Methods	Targets	Range of Detection	LOD	Reference
Colorimetric biosensor	*S. typhimurium*	-	8.59 pM	[174]
Colorimetric biosensor	*Salmonella*	1.8 × 10^1^ to 1.8 × 10^5^ CFU/mL	18 CFU/mL	[175]
Colorimetric biosensor	*Salmonella*	10^2^ to 10^5^ CFU/mL	41 CFU/mL	[176]
SERS biosensor	*S. typhimurium*	3.3 × 10^2^–3.3 × 10^6^ CFU/mL	110 CFU/mL	[177]
Colorimetric biosensor	*S. aureus*	10 to 1 × 10^6^ CFU/mL	2 CFU/mL	[178]
Fluorescent genosensor	*S. aureus*	1 × 10^−17^ to 1 × 10^−11^ mol /L	0.98 × 10^−17^ mol /L	[179]
Fluorescence biosensor	*S. aureus*	10 to 10^6^ CFU/mL	6.9 CFU/mL	[180]
Ratiometric fluorescence biosensor	*S. aureus*	7.9 × 10^0^ to 7.9 × 10^8^ CFU/mL	3 CFU/mL	[181]
Fluorescence biosensor	*S. aureus*	63–6.3 × 10^6^ CFU/mL	25 CFU/mL	[182]
Colorimetric biosensor	*L. monocytogenes*	3.1 × 10^1^ to 3.1 × 10^5^ CFU/mL	3.1 × 10^1^ CFU/mL	[183]
Colorimetric biosensor	*L. monocytogenes*	3.1 × 10^0^ to 3.1 × 10^6^ CFU/mL	3.1 × 10^1^ CFU/mL	[184]
Fluorescence aptasensor	*L. monocytogenes*	68 to 68 × 10^6^ CFU/mL	8 CFU/mL	[185]
Colorimetric biosensor	*S. typhimurium*	1.6 × 10^2^–1.6 × 10^5^ CFU/m3	100 CFU/m^3^	[186]
Fluorescence biosensor	*Salmonella enterica* (*S. enterica*)	6 × 10^1^–6 × 10^7^ CFU/mL	1 CFU/mL	[187]
Fluorescence biosensor	*S. typhimurium*	10–10^7^ CFU/mL	4 CFU/mL	[188]
Colorimetric biosensor	*Salmonella*	5 × 10^1^–5 × 10^5^ CFU/mL	41 CFU/mL	[189]
Ratiometric SERS biosensor	*S. aureus*	10–10^8^ CFU/mL	10 CFU/mL	[190]
Colorimetric biosensor	*S. aureus*	10^−2^ × 10^8^ CFU/mL	2.35 CFU/mL	[191]
Colorimetric-SERS dual-mode aptasensor	*S. aureus*	10^1^ to 10^7^ CFU/mL	0.926 CFU/mL (colorimetric) and 1.561 CFU/mL (SERS)	[192]
SERS biosensor	*S. aureus*	2.15 to 2.15 × 10^5^ CFU/mL	1.0 CFU/mL	[193]
Colorimetric biosensor	*S. aureus*	1 × 10^2^ to 1 × 10^8^ CFU/mL	2 × 10^1^ CFU/mL	[194]
SERS biosensor	*S. aureus*	8.0 to 8.0 × 10^6^ CFU/mL	1.5 CFU/mL	[195]
Fluorescence-enhanced lateral flow biosensor	*S. aureus*	-	5.4 × 10^2^ CFU/mL	[196]
Microfluidic colorimetric biosensor	*E. coli* O157:H7	5 × 10^1^∼5 × 10^6^ CFU/mL	17 CFU/mL	[197]
Colorimetric biosensor	*E. coli* O157:H7	0 to 10^7^ CFU/mL	2 CFU/mL	[198]
SERS biosensor	*E. coli* O157:H7	10 to 10^7^ CFU/mL	2 CFU/mL	[199]
FRET immunosensor	*E. coli* O157:H7	0 to 10^6^ CFU/mL	7 CFU/mL	[200]
Fluorescence biosensor	*E. coli* O157:H7	10 to 10^8^ CFU/mL	17.4 CFU/mL	[201]
Fluorescence biosensor	*E. coli* O157:H7	2.4 × 10^2^ to 2.4 × 10^7^ CFU/mL	2.4 × 10^2^ CFU/mL	[202]
Fluorescence biosensor	*E. coli* O157:H7	500–10^6^ CFU/mL	487 CFU/mL	[203]
Fluorescence biosensor	*V. parahaemolyticus*	10^2^–10^5^ CFU/mL	10^2^ CFU/mL	[204]
Colorimetric-SERS dual-mode	*V. parahaemolyticus*	10^1^–10^5^ CFU/mL	9 CFU/mL (Colorimetric) and 7 CFU/mL (SERS)	[205]

### 3.3. Piezoelectric Transduction

Piezoelectric sensors are signal transduction devices based on the piezoelectric effect, converting biomolecular interactions into measurable frequency, impedance, or acoustic wave changes through the coupling between mechanical deformation and electrical signals in piezoelectric materials [206,207]. Piezoelectric sensors have emerged as critical tools in biosensing due to their high sensitivity, real-time monitoring capability, and label-free operation. Their core strength lies in directly reflecting mass or interfacial property changes during target-analyte binding, enabling quantitative or qualitative analysis [208]. This section focuses on two representative piezoelectric sensing technologies: quartz crystal microbalance (QCM) and surface acoustic wave (SAW) systems.

#### 3.3.1. QCM Transducer

The QCM is a highly sensitive mass-sensing technology leveraging the piezoelectric properties of quartz crystals. Its operation relies on the coupling between the crystal’s mechanical vibration characteristics and surface mass changes. Quartz, an exceptional piezoelectric material, undergoes mechanical resonance under an alternating electric field, with its resonant frequency being mass-dependent. The QCM principle is governed by the Sauerbrey equation, which establishes a linear relationship between surface mass adsorption and resonant frequency shifts. Precise measurement of these frequency variations enables quantitative analysis of adsorbed mass [209]. In foodborne pathogen detection, QCM sensors functionalized with specific biorecognition elements detect bacteria through frequency decreases caused by target binding-induced mass loading, enabling rapid pathogen monitoring [210].

**Figure 9:**
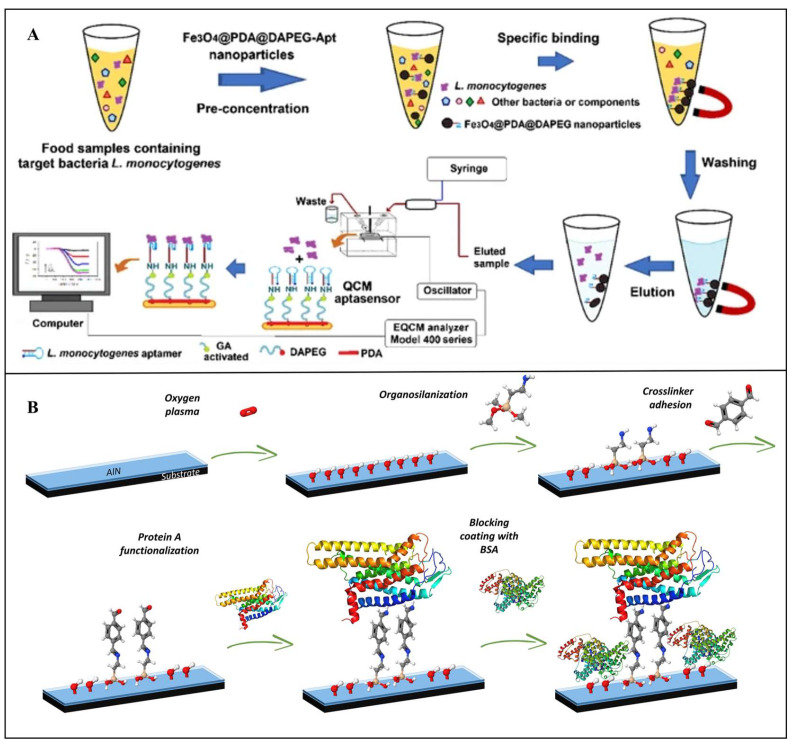
(**A**) Schematic of an aptamer-functionalized QCM sensor coupled with magnetic preconcentration for *L. monocytogenes* detection [211]. Copyright 2022, Springer Nature. (**B**) Schematic of a SAW biosensor for *E. coli* detection [214]. Copyright 2020, Elsevier.

QCM performance in bacterial detection can be enhanced by optimizing surface modification and signal amplification strategies. For instance, Beyazit et al. developed a novel QCM aptasensor for rapid, high-sensitivity detection of *L. monocytogenes*. As shown in Figure 9A, a magnetic preconcentration system using Fe3O4@PDA@DA-PEGnanoparticles and aptamers achieved 91.8% bacterial capture efficiency within 10 min. The aptamer-modified QCM sensor then quantified enriched bacteria, demonstrating a linear range of 1.0 × 10^2^–1.0 × 10^7^ CFU/mL, with an LOD of 148 CFU/mL [211]. Despite its advantages, QCM faces limitations, including nonspecific adsorption on crystal surfaces, complex sample pretreatment, and high instrumentation costs. Future research should prioritize high-performance biorecognition elements, advanced surface functionalization techniques, and miniaturized integrated platforms to expand QCM applications in food safety.

#### 3.3.2. SAW Transducer

SAW sensors are highly sensitive transduction devices based on the propagation characteristics of acoustic waves on piezoelectric materials. They detect target analytes by measuring changes in signal parameters when acoustic wave propagation is perturbed by external physical or chemical interactions. SAW sensors typically consist of a piezoelectric substrate and interdigitated electrodes (IDEs) patterned on its surface. When an alternating voltage is applied to the IDEs, acoustic waves propagate along the substrate surface, with their propagation properties being highly sensitive to surface environmental changes [212,213].

In foodborne pathogen detection, SAW sensors detect target bacteria through surface-functionalized modifications that directly capture pathogens, inducing measurable changes in acoustic wave signals. For example, Lamanna et al. pioneered a flexible and recyclable SAW immunosensor using an aluminum nitride (AlN)-coated polyethylene naphthalate (PEN) substrate for *E. coli* detection. As shown in Figure 9B, the sensor leverages an innovative Protein A/antibody functionalization strategy to achieve rapid pathogen capture. Compared to conventional rigid silicon-based AlN SAW devices, the flexible PEN substrate supports Lamb wave propagation, which enhances sensitivity. The LOD improved from 1.04 × 10^6^ CFU/mL to 6.54 × 10^5^ CFU/mL. Finite element modeling estimated the mass of a single *E. coli* cell (~9 × 10^−13^ g), validating the sensor’s response mechanism to bacterial mass loading [214]. To further advance performance, recent studies have integrated SAW sensors with nanomaterials or microfluidic preconcentration systems. Nanostructured surfaces enhance bacterial capture efficiency, while magnetic preconcentration steps reduce interference from complex food matrices, pushing LODs to single-cell levels [215,216].

## 4. Conclusions and Perspectives

Biosensor technologies have revolutionized the detection of foodborne pathogens by enabling highly specific, sensitive, and rapid analysis across a variety of platforms. This review has systematically summarized the design principles, recognition elements, and transduction mechanisms involved in biosensor-based detection, offering a comprehensive perspective on their potential for improving food safety monitoring.

Despite these promising advancements, several critical challenges continue to hinder the widespread deployment of biosensors in real-world food industry settings. First, high production costs—especially those associated with the synthesis and functionalization of nanomaterials such as MOFs and Au@Ag core–shell nanoparticles—pose significant barriers to scalable manufacturing. Second, biosensor performance often declines in complex food matrices due to nonspecific adsorption, signal interference, or reduced biorecognition stability. Third, many high-performance biosensors still rely on bulky, laboratory-based instrumentation, while portable platforms require further improvement in robustness, anti-interference capability, and long-term operational stability. Furthermore, the lack of standardized validation frameworks and regulatory alignment hampers inter-study data comparability and delays commercialization.

To address these limitations, future biosensor development must advance in several directions. The discovery and engineering of novel recognition elements—such as nanobodies, biomimetic receptors, and machine-learning-optimized aptamers—can improve binding specificity, environmental tolerance, and reduce production complexity. Simultaneously, the integration of multimodal signal transduction, including electrochemical, optical, and piezoelectric methods, can enhance detection reliability through cross-validation. Additionally, intelligent and miniaturized biosensor platforms that incorporate microfluidic chips, flexible electronics, and AI-driven signal interpretation (e.g., deep learning for spectral or impedance data) will be crucial for on-site, high-throughput analysis.

To enhance applicability in low-resource settings, increasing attention has been directed toward the development of biosensors that operate with minimal infrastructure. Electrochemical sensors based on screen-printed electrodes can be coupled with portable or battery-powered potentiostats, while colorimetric and smartphone-assisted fluorescence biosensors allow for instrument-free or low-instrumentation detection, eliminating the need for stable electricity or cold-chain logistics. These features make such platforms well-suited for field deployment and point-of-care testing in remote or under-equipped environments. Environmental and economic sustainability should also be prioritized. The use of biodegradable materials, reusable sensor substrates, and self-driven detection systems—such as those based on phages or CRISPR-Cas technologies—can reduce reagent consumption, operational costs, and environmental impact, aligning biosensor design with green innovation principles.

Importantly, while the commercialization of biosensors remains limited, it is steadily progressing. Notable examples include the 3M™ Molecular Detection System and the bioMérieux VIDAS^®^ platform, both of which have demonstrated successful integration into routine food safety workflows. To facilitate broader adoption, it is essential to address cost-performance trade-offs compared to conventional techniques like PCR and ELISA, streamline sensor fabrication for mass production, and develop robust, ready-to-deploy packaging formats. However, biosensors enhanced with advanced nanomaterials such as MOFs or nanozymes still face challenges in terms of cost-effectiveness, especially when compared to low-cost lateral flow assays. Addressing this will require innovations in green synthesis, the use of earth-abundant precursors, and the development of reusable sensing substrates. Moreover, early-stage collaboration with regulatory agencies, as well as the establishment of unified performance evaluation standards, are critical steps to bridge the gap between academic research and industrial-scale deployment.

In conclusion, continued innovation, cross-disciplinary integration, and commercialization-oriented development will drive biosensors toward becoming indispensable tools for intelligent, precise, and scalable food safety monitoring—ultimately safeguarding the entire supply chain from production to consumption.

## Figures and Tables

**Table 1:** Comparison of major biorecognition elements used in biosensors for foodborne pathogen detection.

Biorecognition Element	Recognition Mechanism	Key Features	Advantages	Limitations
Antibodies	Specific antigen–antibody binding	Y-shaped proteins with high affinity	High specificity; widely used; commercial availability	Poor stability to pH/temperature; high production cost
Aptamers	Target-induced conformational binding via nucleic acid sequences	Synthetic single-stranded DNA/RNA	Chemically stable; easily modified; cost-effective	Structural instability; off-target binding; SELEX selection is time-consuming
Enzymes	Catalytic reaction with target substrate	Biological catalysts	Signal amplification; well-characterized reactions	Sensitive to environmental conditions; short shelf life
Cell Receptors	Natural ligand–receptor interactions (e.g., host–pathogen mimicry)	Membrane or cytosolic proteins/glycoproteins	High biological relevance; specificity to pathogens	Complex structure; low availability; difficult immobilization
MIPs	Template-based molecular imprinting	Synthetic polymeric materials	High stability; low cost; suitable for harsh conditions	Lower selectivity; batch variability; complex preparation
Bacteriophages	Host-specific binding to bacterial surface structures	Viruses that infect specific bacteria	High specificity; self-replicating; can lyse target bacteria	Narrow host range; stability issues; limited commercial availability

## Data Availability

No new data were created or analyzed in this study. Data sharing is not applicable to this article.
